# Genetic Characterization of a Novel Mutant of Peste Des Petits Ruminants Virus Isolated from* Capra ibex* in China during 2015

**DOI:** 10.1155/2016/7632769

**Published:** 2016-02-21

**Authors:** Zixiang Zhu, Xiaocui Zhang, Gulizhati Adili, Jiong Huang, Xiaoli Du, Xiangle Zhang, Pengfei Li, Xueguang Zheng, Xiangtao Liu, Haixue Zheng, Qinghong Xue

**Affiliations:** ^1^State Key Laboratory of Veterinary Etiological Biology, National Foot and Mouth Diseases Reference Laboratory, Key Laboratory of Animal Virology of Ministry of Agriculture, Lanzhou Veterinary Research Institute, Chinese Academy of Agricultural Sciences, Lanzhou, Gansu 730046, China; ^2^Institute of Veterinary Medicine, Xinjiang Academy of Animal Science, Urumqi, Xinjiang 830000, China; ^3^Wildlife Epidemics and Epidemic Sources Monitoring Center of Xinjiang Uyghur Autonomous Region, Urumqi, Xinjiang 830000, China; ^4^China Animal Health and Epidemiology Center, Qingdao, Shandong 266000, China; ^5^China Institute of Veterinary Drugs Control, Beijing 100000, China

## Abstract

Peste des petits ruminants virus (PPRV) is the causative agent of peste des petits ruminants (PPR). The spread of PPR often causes severe economic losses. Therefore, special attention should be paid to the surveillance of PPR emergence, spread, and geographic distribution. Here we describe a novel mutant of PPRV China/XJBZ/2015 that was isolated from* Capra ibex* in Xinjiang province in China 2015. The sequence analysis and phylogenetic assessment indicate that China/XJBZ/2015 belongs to lineage IV, being closely related to China/XJYL/2013 strain. Interestingly, the V protein sequence of China/XJBZ/2015 showed lower homology with other Chinese PPRVs isolated during 2013 to 2014 (94%~95%), whereas it shared 100% identity with three Tibet strains isolated in China 2007. The 3′ UTR, V gene, and C gene were determined to be highly variable. Besides, 29 PPR genomic sequences available in GenBank were analyzed in this study. It is the first time to use PPRV genomic sequences to classify the different lineages which confirmed the lineage clustering of PPRVs using N gene 255 bp fragments and F gene 322 bp fragments. In conclusion, our findings indicate that the PPRVs continue to evolve in China, and some new mutations have emerged.

## 1. Introduction

Peste des petits ruminants (PPR) is an important infectious disease of small ruminants [1]. Peste des petits ruminants virus (PPRV) is the etiological agent of PPR belonging to genus* Morbillivirus* of the family of Paramyxoviridae [2]. PPRV is an enveloped, negative-sense single-stranded RNA virus with the genome of about 15948–15957 nucleotides (nt) in length. The viral genome encodes six structural proteins including nucleocapsid (N), phosphoprotein (P), matrix (M), fusion (F), haemagglutinin (H) and large polymerase (L), and two nonstructural proteins, C and V [3].

PPR is reported in several countries in Africa, Arabian Peninsula, Middle East, and Asia [1, 4]. It is estimated that about 63% small ruminant populations are at risk of PPR by the Food and Agriculture Organization, especially those from Southern Africa, Southern Europe, Middle East, central Asia, and China [5]. The potential threat of PPR on small ruminant production has resulted in PPRV becoming a global animal health concern. The control and eradication of PPR have emerged as an urgent mission for many countries [6].

The PPRVs exist as one serotype, whereas they present the characteristics of high genetic variability. The viral genome of PPRV consistently mutates during the evolution and spread of the viruses [7]. PPRVs have been classified into four lineages based on the molecular characteristics of different virus strains (lineages I, II, III, and IV) [8, 9]. The strains isolated in Western Africa mainly belong to lineages I and II [5]. Cocirculation of lineage II PPRV along with lineages III and IV is reported in Eastern Africa, and lineage II has been circulating in Central Africa [10]. The PPRVs circulating in the Middle East and Asia are mainly lineage IV strains [5]. More viruses are isolated in different parts of the world and the geographic distribution of PPRVs has become more complicated. In China, most of the strains are included in lineage IV. A 6-nucleotide insertion in the 5′ UTR of F gene was found in a China/XJYL/2013 PPR strain in 2013 and a China/BJ/2014 strain in 2014 [11, 12]. Several lineage II strains have been also observed in China in recent years [13]. It indicates that the current evolutionary and epidemiological status of PPRVs in China is also complicated. The present study isolated a new PPRV strain from ibex (*Capra ibex*) in China 2015. The whole-genome was determined, which will contribute to an improved understanding of PPRV epidemiology for the China Himalayan region through whole-genome and sequence analysis from an isolate taken from an infected wild species of caprine.

## 2. Materials and Methods

### 2.1. Clinical Disease

In the Bazhou region of Xinjiang province, China, 38 ibex died in unusual circumstances from January to February, 2015. It seemed to be PPR according to clinical signs and symptoms including fever, lassitude, anorexia, pneumonia, and diarrhea; the mucopurulent discharge was clearly observed in the eyes and nose of the suspicious ibex, and congested conjunctivae, necrotic lesions on lips and gums were shown. Xinjiang Academy of Animal Science cooperated with China Animal Health and Epidemiology Center to investigate the mortalities in ibex. Lungs, lymph nodes, and swab samples were collected from the suspected ibex, and the postmortem lesion signs were recorded. The postmortem examination revealed congestion in different lobes of lungs. The clinical signs and postmortem examination led to the suspicion of PPR. All the collected samples were kept at −80°C for the subsequent virus isolation.

### 2.2. Virus Isolation

The tissue samples were obtained from the infected animal in this outbreak and African green monkey (Vero) cells were used for PPRV isolation. The homogenized tissues were centrifuged for 10 min at 10,000 rpm, 4°C, to remove the debris. The supernatant was filtered through a 0.22 *μ*m membrane. The obtained virus was incubated on previously prepared monolayer Vero cells. The inoculum was removed and replaced with DMEM plus 2% fetal bovine serum after 2 h adsorption at 37°C. The cytopathic effect (CPE) was observed and recorded every day after virus incubation. The mock-infected Vero cells were used as a negative control. All ethics, field, and experimental work have been approved and performed in compliance with the standard guidelines of the Gansu Animal Experiments Inspectorate and the Gansu Ethical Review Committee (License number SYXK [GAN] 2010-003).

### 2.3. RNA Extraction and PPRV Detection

The tissue homogenates and prepared PPRV-infected cell culture were used for RNA extraction, the total RNA was extracted with Trizol reagent (Invitrogen) according to the instructions provided by the manufacturer, and the total RNA was resuspended in 25 *μ*L of nuclease free water. Five microliters of extracted RNA was used to synthesize the cDNA fragments. The reverse transcriptase-polymerase chain reaction (RT-PCR) for PPRV detection was performed using PPRV partial N gene primers that were reported previously [14, 15]. The RT-PCR assay was performed as previously described by Kerur et al. [15]. The amplified PCR products were resolved on a 1.5% agarose gel stained with 0.5 *μ*g/mL ethidium bromide.

### 2.4. Sequence Cloning and Sequencing

The extracted RNA from one collected positive tissue sample was used for cDNA synthesis, and the obtained cDNA were used directly for genome determination using 14 pairs of primers from the previously published article [11]. The amplified fragments were cloned into the pGEM-T easy vector (Promega) for genome sequencing. The termini of the China/XJBZ/2015 were determined by rapid amplification of cDNA ends (RACE) as previously described [16].

### 2.5. Sequence Analysis

The sequence identity was analyzed using the MegAlign project of the DNAStar software (DNASTAR, Inc., USA). The PPRVs genome sequences and partial genes of Chinese strains available in GenBank were collected and used for sequence analysis. The phylogenetic trees were constructed using the clustalx1.83 and MEGA5.1. Phylogenetic analyses were performed using neighbor-joining method and the Kimura-2-parameter nucleotide substitution model in MEGA5.1 (http://www.megasoftware.net/). The numbers of bootstrap replicates were set as 1000.

## 3. Results

### 3.1. Virus Replication in Cell Cultures

**Figure 1:**
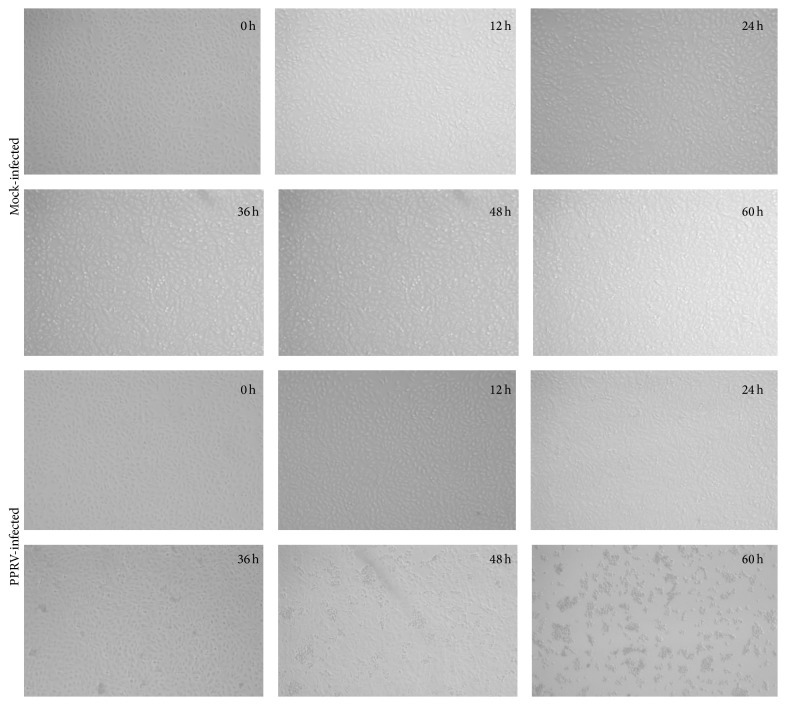
PPRV-infected CPE cells. Vero cells were infected with China/XJBZ/2015 strain or mock-infected for 0, 12, 24, 36, 48, or 60 h; the CPE were observed at the indicated time points. In China/XJBZ/2015-infected cells, significant cell disruption appeared after 48 hpi.

The detection of the PPR-infected samples was determined by RT-PCR and virus isolation. The PPR-positive sample generated clear CPE in Vero cells at 48 h after inoculation (hpi), and the infected cells showed more significant cell disruption and partial focal detachment at 60 hpi (Figure 1). The RT-PCR detection of the virus-incubated cell culture and the CPE results confirmed the successful isolation of this PPR virus. It was the first time to isolate a PPRV strain from ibex in Xinjiang in China. The isolated virus was named as peste des petits ruminants virus China/XJBZ/2015 and maintained at −80°C.

### 3.2. Full-Length Genomic Sequence of China/XJBZ/2015

The viral genome of the China/XJBZ/2015 virus strain was determined by RT-PCR. Fourteen overlapping fragments were successfully amplified. The genome termini were also determined. The results indicate that the genome of China/XJBZ/2015 is 15954 nt in length. The genomic organization of China/XJBZ/2015 is shown in Table 1. The gene order of the China/XJBZ/2015 was determined as 3′-N-P/C/V-M-F-H-L-5′ flanked by a 3′ genomic promoter of 107 nt and a 5′ antigenomic promoter of 109 nt. The genome sequence of China/XJBZ/2015 has been deposited into GenBank with the accession number of KT633939.

### 3.3. Genetic Assessment of PPRV

The nucleotide and amino acid sequences of the different genes of China/XJBZ/2015 were compared with other PPRV reference strains available in GenBank (Table 2). The results revealed that the nucleotides sequences and amino acid sequences of L, M, and F genes were comparatively conservative, while other regions were divergent (Tables 3 and 4). Of these viral genes, L exhibited the most conservative amino acid identity (95.2%–99.8%) comparing with the listed strains, and M and F showed 94.6%–99.1% and 93.4%–98.7% identity, respectively. The highly variable P, V, and C proteins showed 83.9%–99.8%, 79.5%–100%, and 74.6%–100% amino acid identity, respectively (Table 3). Besides, it was observed that the nucleotide similarity of both the 3′ genomic UTR and 5′ genomic UTR was variable (88.8%–100% and 85.3%–100%) (Table 4). At the genomic nucleotide level, 29 PPR genomic sequences available in GenBank (with 8 Chinese strains and 21 foreign strains) were compared with the genome of China/XJBZ/2015; the result indicated that China/XJBZ/2015 shares extremely high homology with the Chinese strains isolated from 2013 and 2014 (99.1%–99.4%), with the highest homology of 99.4% with China/XJYL/2013. Although PPRV has high genetic variability, this result confirmed the stable evolutionary status of PPRV as it was previously reported that PPRV does not undergo rapid genetic changes [16–18]. Interestingly, it was observed that the V protein sequence of China/XJBZ/2015 was similar to the strains isolated in Tibet in 2007 (100% homology) and showed lower amino acid similarity with the strains isolated from 2013 and 2014 (94.0%–95.0%) (Table 3). F protein sequence showed 98.5%–98.7% homology with the other Chinese strains isolated from 2013 and 2014, and 8 amino acids in the F protein of China/XJBZ/2015 were different with other Chinese strains isolated from 2013 and 2014. The 3′ UTR of China/XJBZ/2015 exhibited 98.1%–100% nucleotide identity with 4 Chinese isolates from 2014, and 97.2% with China/XJYL/2013. The 5′ UTR of China/XJBZ/2015 shared 100% nucleotide identity with 3 Chinese strains isolated 2014 and China/XJYL/2013 and shared 97.2% with 3 Chinese strains isolated in Tibet 2007 (Table 4). It indicates the different genetic characteristics of China/XJBZ/2015 comparing with other Chinese PPR strains.

### 3.4. Phylogenetic Tree

**Figure 2:**
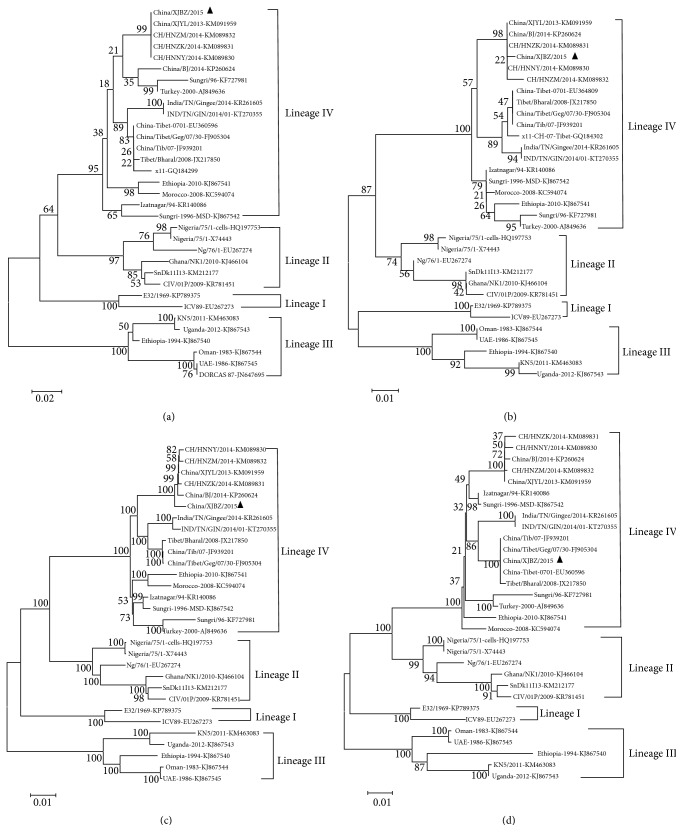
Phylogenetic analysis of the PPRVs using the whole-genome and multigene sequences data. (a) Phylogenetic tree for the 255 bp N gene of PPRV; (b) phylogenetic tree for the 322 bp F gene of PPRV; (c) phylogenetic tree for the viral whole-genome of the PPRVs; and (d) phylogenetic tree for the V gene of PPRV. The phylogenetic trees were constructed using the clustalx1.83 and MEGA5.1. Phylogenetic analyses were performed using neighbor-joining method and the Kimura-2-parameter nucleotide substitution model in MEGA5.1 (http://www.megasoftware.net/). The numbers of bootstrap replicates were set as 1000.

The consensus phylogenetic trees of PPRVs based on the lineage specific 322 bp F gene sequences [8] and the variable region of the N genes (255 bp) [19, 20] were constructed to classify the different lineages. Four lineages (lineages I, II, III, and IV) were clearly divided and China/XJBZ/2015 was clustered into lineage IV (Figure 2). Besides, the phylogenetic trees based on the viral complete genome sequences and V gene sequences were also constructed. The phylogenetic tree based on the complete genome sequences divided the PPRV strains into four lineages as using N and F gene fragments. The phylogenetic tree based on V gene coding region also clearly divided the four different lineages; however, China/XJBZ/2015 was clustered into a different branch comparing with other three clustering methods. It also confirmed the different genetic characteristics of China/XJBZ/2015.

## 4. Discussion

PPRV is genetically highly variable like other RNA viruses [5]. Perhaps, the apparent expansion of PPR in North and East Africa and in Asia has occurred despite the existence of an available vaccine [13, 14, 21–23]. The viral genome and partial fragment sequencing of PPRVs has been widely performed to determine the spread status and to analyse the genetic variability. Despite work on PPR over many years, there are relatively few PPRV sequences available now, and the evolutionary information and spread status of many geographic regions and countries remain unclear due to the limited sequences; therefore, any interpretation of the molecular epidemiology needs to be approached with caution. There are only 29 PPRV genome sequences available in GenBank at the time of the writing of this paper. Therefore, more PPRV genomes information is essential for further research aimed at investigating the distribution of PPRVs and developing strategies for prevention and control of PPR.

In this study, we isolated a PPRV strain from ibex in Xinjiang province in China 2015 by performing CPE assay and viral genome sequencing. The sequence identity analysis results indicated that China/XJBZ/2015 includes some new mutations comparing with previous Chinese PPRVs. It implied that PPRVs have evolved in ibex in China. The alignment of the different genes of the all used Chinese strains indicates that the 3′ UTR, V gene, and C gene are the high variable regions. Possibly, these genes can be used as the potential evolutionary markers for analysis of the mutation status of Chinese PPRV strains.

The genome sequence identity indicated that China/XJBZ/2015 shares high homology with other five Chinese strains isolated during 2013 to 2014 and shares highest homology with China/XJYL/2013, whereas the V protein sequence of China/XJBZ/2015 is significantly different from the other five newly reported Chinese strains, and interestingly, it shares 100% homology with other three Chinese Tibet strains isolated in 2007. Bazhou region is situated at the south Xinjiang province in China. This region includes the second largest prairie of China. A wide range of wild animals including ibex, argali, wild yak, and deer share pasture and water resources with the resident livestock. In addition, Bazhou is bounded on the south by the Tibet of China. Migratory wildlife populations in Xinjiang and Tibet may create a circumstance for the meeting of different PPRV strains. Whether a gene drift or a reassortment resulted in the mutation of China/XJBZ/2015 remains unknown. The 3′ UTR regions are also variable in the six Chinese PPRV strains isolated during 2013 to 2015. A 6-nucleotide insertion was firstly observed in the 5′ UTR of the F gene of China/XJYL/2013 in 2013 [11], and after that, almost all of Chinese lineage IV PPRVs include this insertion. It has been reported that F gene is highly conserved between different PPRV strains [14, 18]; our results demonstrated the conservative characteristic of F gene in Chinese strains isolated in recent three years. However, eight amino acids in the F protein of China/XJBZ/2015 were different with other Chinese strains isolated from 2013 to 2014. Five of the amino acids are identical with some other reported PPRV strains, and another three amino acid substitutions are new mutations. F protein is believed to disrupt the target cell membrane, hence inducing the virus-cell and cell-cell fusion [24]. The three amino acid substitutions appeared at the residue of 109, 205 and 206 in F protein. Whether these residues are involved in the regulation of fusion process remains unclear [25]. China/XJBZ/2015 resulted in a high mortality to the affected ibex; whether these amino acids substitutions enhanced the attachment or fusion of the virus particle with the ibex cells and increased the pathogenicity should be further studied. High mortality has been observed in Sindh Ibex affected by PPRV in a national park in Pakistan [26]; there was about 38 ibex deaths during the period of 1 month in this study. It indicates PPR may threaten the huge population of ibex. Therefore, more attention should be paid to the surveillance of PPR emergence in ibex.

The analysis of PPRV population variation has been constructed by aligning the partial F gene or N gene [8, 20]. In accordance with the previous studies, all the strains were clustered into four different lineages. The phylogenetic trees constructed based on 322 bp F gene and 255 bp N gene are similar to the phylogenetic tree constructed based on the whole-genome sequences. It is the first time to use PPRV genomic sequences to classify the different lineages, and it confirmed the lineage clustering of PPRVs using N gene 255 bp fragments and F gene 322 bp fragments.

In conclusion, a novel mutant of PPRV has been isolated from ibex in China 2015, and the viral genes analysis results suggest that the PPRVs in China continue to evolve. PPR may threaten the huge population of ibex in Xinjiang province in China. Due to the mutation of PPRV, it is desirable to present more virus genome sequences to unravel the evolutionary path, geographic distribution, and spread status in different regions.

## Figures and Tables

**Table 1:** The genomic organization of China/XJBZ/2015 and the sequence identity blast with the available sequences in GenBank.

Gene	Nucleotides location	Amino acids number	Nucleotide identity (%)		Amino acid identity (%)	
			Minimum	Maximum	Minimum	Maximum
3′ UTR	1–107	—	88.8 Ethiopia 1994	100 CH/HNZK/2014	—	—
N	108–1685	525	88.7 Uganda 2012	99.7 China/XJYL/2013	92.2 DORCAS_87	99.6 China/XJYL/2013
P	1807–3336	509	88.0 Ethiopia 1994	99.9 China/XJYL/2013	83.9 Ethiopia 1994	99.8 China/XJYL/2013
V	1807–2702	298	87.5 Ethiopia 1994	100 China/Tibet/Geg/07-30	79.5 Ethiopia 1994	100 China/Tibet/Geg/07-30
C	1829–2362	177	84.5 Ethiopia 1994	100 China/XJYL/2013	74.6 Ethiopia 1994	100 China/XJYL/2013
M	3438–4445	335	89.2 KN5/2011	98.9 China/XJYL/2013	94.6 Sungri/96	99.1 China/XJYL/2013
F	5532–7172	546	88.1 Uganda 2012	98.5 China/XJYL/2013	93.4 Uganda 2012	98.7 China/XJYL/2013
H	7332–9161	609	87.5 Uganda 2012	99.5 China/XJYL/2013	89.5 Uganda 2012	99.7 CH/HNZM/2014
L	9294–15845	2183	89.2 Uganda 2012	99.7 China/XJYL/2013	95.2 Uganda 2012	99.8 China/XJYL/2013
5′ UTR	15846–15954	—	85.3 Ethiopia 1994	100 CH/HNZK/2014	—	—
Genome	1–15954	—	87.2 KN5/2011	99.4 China/XJYL/2013	—	—

**Table 2:** The information of the PPRV reference strains used in this study.

Accession	Isolate name	Country, collection date	Lineage	Host	Genome length
KT633939	China/XJBZ/2015	China, 2015-February	IV	*Capra ibex*	15954
KP260624	China/BJ/2014	China, 2014-August	IV	Goat milk	15954
KM089831	CH/HNZK/2014	China, 2014-May	IV	Goat	15957
KM089832	CH/HNZM/2014	China, 2014-May	IV	Goat	15954
KM089830	CH/HNNY/2014	China, 2014-May	IV	Goat	15954
KM091959	China/XJYL/2013	China, 2013-November	IV	Goat	15954
JX217850	Tibet/Bharal/2008	China, 2008	IV	Wild bharal	15948
JF939201	China/Tib/07	China, 2007-Dec	IV	Goat	15948
FJ905304	China/Tibet/Geg/07-30	China, 2007-August	IV	Goat	15948
GQ184299	x11 (N-M-F-H)	China, 2007-August	IV	Goat	—
EU360596	China/Tibet/0701(N-P/C/V-M-F-H-L)	China, 2007	IV	Goat	—
KR261605	India/TN/Gingee/2014	India, 2014-September	IV	Goat	15948
KT270355	IND/TN/GIN/2014/01	India, 2014-September	IV	Goat	15942
KF727981	Sungri/96	India, 1996	IV	Goat	15948
KR140086	Izatnagar/94	India, 1994	IV	Goat	15948
KJ867541	Ethiopia 2010	Ethiopia, 2010	IV	Goat	15948
KC594074	Morocco 2008	Morocco, 2008	IV	Alpine goat	15948
AJ849636	Turkey 2000	Turkey, 2000	IV	*Ovis aries*	15948
KJ867542	Sungri 1996 MSD (The Netherlands)	India, 1996	IV	Vaccine Strain	15948
KJ867543	Uganda 2012	Uganda, 2012	III	Goat	15948
KM463083	KN5/2011	Kenya, 2011-May	III	Goat	15948
KJ867540	Ethiopia 1994	Ethiopia, 1994	III	Goat	15948
JN647695	DORCAS_87 (N protein)	Oman, 1987	III	Unknown	—
KJ867545	UAE 1986	United Arab Emirates, 1986	III	Dorcas gazelle	15948
KJ867544	Oman 1983	Oman, 1983	III	Goat	15948
X74443	Nigeria/75/1	Nigeria, 1975	II	Caprine goat	15948
HQ197753	Nigeria/75/1	Nigeria, 1975	II	Rescue in Vero cells	15948
EU267274	Ng76/1	Nigeria, 1976	II	Goat	15948
KR781451	CIV/01P/2009	Cote d'Ivoire, 2009-July	II	Goat	15948
KJ466104	Ghana/NK1/2010	Ghana, 2010	II	Sheep	15948
KM212177	SnDk11I13	Senegal, 2013-March	II	Goat	15948
KP789375	E32/1969	Senegal, 1969	I	Goat	15948
EU267273	ICV89	Cote d'Ivoire, 1989	I	Goat	15948

**Table 3:** The amino acids identity of the different proteins of China/XJBZ/2015 compared with the reference strains used in this study.

Lineages	Virus strain	N	P	V	C	M	F	H	L
IV	China/BJ/2014	98.9	99.6	94.6	99.4	99.1	98.7	99.5	99.5
	CH/HNZK/2014	99.4	98.8	94.0	98.9	99.1	98.7	99.3	99.6
	CH/HNZM/2014	99.6	99.6	95.0	100	99.1	98.5	99.7	99.5
	CH/HNNY/2014	99.6	99.8	94.6	98.9	99.1	98.5	99.2	99.1
	China/XJYL/2013	99.6	99.8	94.6	100	99.1	98.7	99.5	99.8
	Tibet/Bharal/2008	98.3	96.3	99.3	93.2	98.8	97.6	98.2	98.6
	China/Tib/07	98.3	96.5	100	94.4	98.8	97.6	98.2	98.6
	China/Tibet/Geg/07-30	98.6	96.5	100	94.4	98.8	97.4	98.2	98.7
	x11	98.3	—	—	—	98.2	97.6	98.2	—
	China/Tibet/0701	98.5	96.5	100	94.4	98.6	97.4	98.2	98.6
	India/TN/Gingee/2014	97.5	94.9	95.0	94.9	99.1	97.4	97.0	98.7
	IND/TN/GIN/2014/01	97.7	95.1	95.0	94.9	99.1	97.4	97.4	98.7
	Sungri/96	96.4	94.1	92.3	91.0	94.6	94.7	95.1	95.9
	Izatnagar/94	97.7	97.8	96.6	97.2	98.5	98.0	97.9	98.9
	Ethiopia 2010	96.8	95.9	95.6	95.5	97.9	97.1	96.7	98.4
	Morocco 2008	97.1	96.7	95.6	96.0	98.2	97.1	97.2	98.5
	Turkey 2000	97.5	96.9	95.3	93.8	96.7	97.3	98.2	98.7
	Sungri 1996 MSD	97.5	97.6	96.6	97.2	97.6	97.8	97.2	98.7

III	Uganda 2012	92.4	85.7	82.6	80.8	96.1	93.4	89.5	95.2
	KN5/2011	92.4	85.5	82.2	80.2	95.8	93.4	89.7	95.2
	Ethiopia 1994	93.7	83.9	79.5	74.6	95.8	93.8	91.0	96.0
	DORCAS_87	92.2	—	—	—	—	—	—	—
	UAE 1986	92.4	87.0	83.9	81.4	95.8	94.3	90.5	96.0
	Oman 1983	92.2	87.0	83.9	81.4	95.8	94.1	90.5	96.1

II	Nigeria/75/1	94.7	91.0	89.9	90.4	97.3	96.9	92.8	97.7
	Nigeria/75/1 (cell strain)	94.5	91.0	89.9	90.4	97.0	96.9	92.9	97.7
	Ng76/1	93.9	90.0	82.9	89.3	97.9	97.6	93.1	97.6
	SnDk11I13	94.7	88.6	85.9	85.9	97.6	96.7	92.9	97.3
	CIV/01P/2009	94.5	87.8	84.9	85.3	97.0	96.7	92.3	97.3
	Ghana/NK1/2010	94.5	89.0	85.9	86.4	97.3	96.7	92.8	97.3

I	E32/1969	95.0	89.0	86.2	88.7	97.3	96.2	91.8	96.9
	ICV89	94.1	86.4	77.9	85.3	97.0	93.6	91.0	96.5

**Table 4:** The nucleotide identity of the regions of China/XJBZ/2015 compared with the reference strains used in this study.

Virus strain	3′ UTR	N	P	V	C	M	F	H	L	5′ UTR	Genome	F-322	N-255
China/BJ/2014	100	98.9	99.7	97.0	99.8	98.9	98.4	99.3	99.5	99.1	99.1	99.7	97.3
CH/HNZK/2014	100	99.7	99.6	96.8	99.4	98.9	98.5	99.4	99.6	100	99.3	99.7	100
CH/HNZM/2014	98.1	99.6	99.8	97.1	100	98.7	98.3	99.3	99.5	100	99.2	99.1	100
CH/HNNY/2014	98.1	99.7	99.7	96.9	99.6	98.9	98.4	99.3	99.4	100	99.2	99.7	100
China/XJYL/2013	97.2	99.7	99.9	97.2	100	98.9	98.5	99.5	99.7	100	99.4	99.7	100
Tibet/Bharal/2008	97.2	97.8	97.3	99.8	96.3	97.3	96.5	97.5	97.9	97.2	96.9	97.2	96.1
China/Tib/07	97.2	97.8	97.4	100	96.6	97.4	96.6	97.7	97.9	97.2	97.0	97.2	96.5
China/Tibet/Geg/07-30	97.2	97.8	97.4	100	96.6	97.4	96.5	97.7	97.9	97.2	97.0	97.2	96.5
x11	—	97.6	—	—	—	97.2	96.4	97.5	—	—	—	97.2	95.3
China-Tibet-0701	—	97.8	97.4	100	96.6	97.6	96.5	97.6	97.9	—	—	97.2	96.5
India/TN/Gingee/2014	94.4	97.5	96.8	97.7	96.6	97.3	96.3	96.6	97.3	94.5	96.4	96.9	94.9
IND/TN/GIN/2014/01	95.3	97.5	96.9	97.7	96.6	97.0	96.1	96.4	97.2	94.5	96.2	96.9	94.9
Sungri/96	96.3	96.5	96.3	96.0	95.3	95.1	94.6	95.1	96.2	92.7	95.2	96.0	92.2
Izatnagar/94	98.1	97.5	98.2	98.3	97.9	97.9	97.1	97.5	97.9	93.6	97.1	96.6	93.3
Ethiopia 2010	96.3	96.8	97.0	97.2	96.6	96.8	95.7	96.1	97.0	95.4	95.9	97.5	93.7
Morocco 2008	96.3	96.9	97.3	97.4	97.2	97.3	96.2	96.4	97.1	96.3	96.1	97.5	93.3
Turkey 2000	97.2	97.0	97.3	97.3	96.8	96.1	96.0	96.4	97.4	93.6	96.4	96.6	94.5
Sungri 1996 MSD	96.3	97.5	98.1	98.2	97.9	97.7	97.0	97.2	98.0	96.3	97.0	97.8	92.9
Uganda 2012	89.7	88.7	89.3	89.3	88.0	91.7	88.1	87.5	89.2	87.2	88.5	87.9	80.8
KN5/2011	89.7	88.8	89.3	89.2	87.8	89.2	88.0	87.8	89.3	87.2	87.2	88.5	81.2
Ethiopia 1994	88.8	90.6	88.0	87.5	84.5	90.0	89.4	88.8	97.0	85.3	88.3	89.1	83.1
DORCAS_87	—	89.3	—	—	—	—	—	—	—	—	—	—	80.4
UAE 1986	89.7	89.4	89.8	90.4	89.1	89.6	89.4	88.0	90.6	88.1	88.3	90.1	80.4
Oman 1983	90.7	89.2	89.8	90.4	89.0	89.6	89.2	87.9	90.5	88.1	88.2	90.1	80.4
Nigeria/75/1	92.5	93.5	93.0	93.8	93.1	94.3	94.0	91.7	94.1	91.7	92.6	92.5	87.1
Nigeria/75/1 (cell strain)	92.5	93.3	93.0	93.8	93.1	94.2	94.0	91.8	94.1	91.7	92.6	92.9	87.1
Ng76/1	97.2	93.1	92.6	93.0	91.8	94.3	94.3	92.0	94.1	91.7	92.5	93.8	85.9
SnDk11I13	93.5	92.9	91.0	91.5	89.9	92.8	92.7	90.7	93.1	92.7	91.3	91.9	88.2
CIV/01P/2009	93.5	92.6	91.0	91.4	89.5	92.7	92.7	90.4	92.9	90.8	91.1	91.3	88.2
Ghana/NK1/2010	93.5	92.9	91.4	91.7	90.1	92.9	92.4	90.9	92.9	95.4	91.2	91.9	87.8
E32/1969	93.5	92.5	92.1	91.5	91.2	92.8	91.7	90.0	92.0	89.9	90.4	89.8	86.3
ICV89	94.3	91.5	90.3	89.9	88.8	91.7	89.9	89.2	91.0	84.4	89.1	88.8	83.9
